# Evaluation of MUC1, MUC2, MUC5AC, and MUC6 Expression Differences in Lung Adenocarcinoma Subtypes by Using a Final Immunoreactivity Score (FIRS)

**DOI:** 10.5146/tjpath.2022.01593

**Published:** 2023-01-15

**Authors:** Melek Buyuk, Yasemin Ozluk, Dogu Vurallı Bakkaloglu, Berker Ozkan, Pinar Fırat, Dilek Yılmazbayhan

**Affiliations:** Department of Pathology, Istanbul Faculty of Medicine, Istanbul University, Istanbul, Turkey; Department of Thoracic Surgery, Istanbul Faculty of Medicine, Istanbul University, Istanbul, Turkey; Department of Pathology, Koc University School of Medicine, Istanbul, Turkey

**Keywords:** Lung adenocarcinoma, Mucin expression, Immunohistochemistry, MUC1, Immune reactivity score

## Abstract

***Objective:*** Lung adenocarcinomas are divided into acinar, lepidic, papillary, micropapillary, and solid predominant subtypes according to the current World Health Organization (WHO) classification. We designed this retrospective study to demonstrate profiles of MUC expression (MUC1, MUC2, MUC5AC, and MUC6) of different histologic patterns within the same tumor among pulmonary adenocarcinomas and investigate correlations of MUC expression with clinicopathologic features.

***Material and Method:*** We analyzed the expression of mucins (MUC1, MUC2, MUC5AC, and MUC6) in a series of 99 resected lung adenocarcinomas, which included a total of 193 patterns (71 acinar, 30 lepidic, 25 papillary, 20 micropapillary, 34 solid and 13 mucinous) and calculated a final immune reactivity score (FIRS) per tumor.

***Results:*** MUC1 IRS scores were significantly higher in lepidic and solid patterns compared with mucinous patterns (p=0.013). MUC2 expression was seen only in three cases (1 acinar, 2 mucinous). MUC5AC and MUC2 expression was more common in mucinous patterns (p<0.001 and p=0.028, respectively). MUC6 expression was only detected in seven patterns and the expression was weak. No significant difference was seen among histologic patterns for the staining scores of MUC6. Mucinous adenocarcinoma differed from other histologic subtypes regarding MUC1 and MUC5AC expression. Mucinous adenocarcinoma showed less MUC1 expression with lower IRS scores and higher MUC5AC expression. Tumor size (p=0.006), lymphatic invasion (p=0.018), vascular invasion (p=0.025), perineural invasion (p=0.019), MUC1 IRS scores (p=0.018), and MUC1 IRS scores >8.5 (p=0.018) were significant predictors for lymph node metastasis.

***Conclusion:*** An alternative scoring for MUC1 can be used as a predictor for lymph node metastasis regardless of the histologic subtype.

## INTRODUCTION

In the respiratory system, mucus acts as a protective barrier against external factors such as pathogens, chemical agents, and dust particles in the air. In the tracheobronchial epithelium, mucins are synthesized by goblet cells in the surface epithelium and mucous cells in the submucosal glands (1). Mucins are categorized into two groups, membrane-dependent and secretory (gel-forming mucins) (2). MUC1 is a membrane-dependent mucin, whereas others (MUC2, MUC5AC, and MUC6) are gel-forming. Cancer cells, especially in adenocarcinomas, express aberrant forms or amounts of mucins. Cancer cells may use mucins for protection from adverse growth conditions and to control the local molecular microenvironment during invasion and metastasis (3).

MUC1 is normally expressed on the apical borders of epithelial cells, which is called polarized expression, in many tissues including the lung (4–7). MUC1 expression in cancer cells has been shown on the apical border and the lateral cell membrane and in cytoplasm (depolarized staining). MUC1 overexpression enhances the invasion capacity of cancer cells by inhibiting E-cadherin-mediated cell-cell adhesion and integrin-mediated cell adhesion to extracellular matrix components (8,9). Also, MUC1 inhibits cytotoxic T lymphocyte-tumor cell interaction (10). Several studies have been performed on adenocarcinomas and expression abnormalities of mucin glycoproteins because mucins are known to be present in glandular epithelial cells (11–14). Among the mucins, high levels of MUC1 expression in particular are significantly associated with poor prognosis. High expression of MUC1 was associated with the presence of axillary lymph node metastases in breast carcinoma (11) and similarly, high expression of MUC1 was related to a significantly shorter overall survival (OS) in sinonasal adenocarcinomas (12). In gastric and pancreatic carcinomas, MUC1 overexpression and its association with poor prognosis have been well documented (13,14).

Lung adenocarcinomas are heterogeneous groups of tumors and have different histologic patterns (lepidic, acinar, papillary, micropapillary, solid, fetal, and enteric). Invasive mucinous adenocarcinoma and colloid adenocarcinoma also are additional distinctive patterns (15). MUC1 overexpression or depolarized expression also acts as a poor prognostic parameter in lung cancer (16–20). MUC1 is overexpressed in lung cancer, making it an excellent target for immunotherapy. Several clinical trials of MUC1 vaccines in non-small cell lung cancer (NSCLC) have also been reported (21,22).

Previous studies have shown specific mucin expression profiles (overexpression MUC5AC and MUC6) for invasive mucinous carcinoma (formerly known as mucinous bronchioloalveolar adenocarcinoma) (23–27).

However, to the best of our knowledge, differences in the expression profiles of MUCs among different histologic patterns of lung adenocarcinomas have not yet been published. With this aim, we designed this retrospective study to demonstrate profiles of MUC expression (MUC1, MUC2, MUC5AC, and MUC6) of different histologic patterns within the same tumor among pulmonary adenocarcinomas and investigate correlations of MUC expression with clinicopathologic features. In this retrospective study, we tried to answer the following questions:

1. Are there any differences between histologic subtypes of pulmonary adenocarcinomas regarding MUC expression?
2. Is the profile of MUC expression related to any clinicopathologic feature?
3. Since adenocarcinomas are heterogeneous, how should we evaluate MUC1 staining within different patterns of the same tumor?
4. Which parameters are predictors for lymph node metastasis?

## MATERIALS and METHODS

This study was approved by the Clinical Research Ethics Committee of the Istanbul University Faculty of Medicine (file number: 2012/1729-1289).

### Patients

We retrospectively evaluated 99 resection materials of preoperatively untreated lung adenocarcinomas with available paraffin blocks between January 2007 and December 2013. Patients with distant metastasis were not included. Histopathologic and clinical data were retrieved from the pathology reports and clinical records.

### Histopathologic Evaluation

All archived hematoxylin-eosin–stained tumor slides were re-examined by two pathologists (DY, an experienced pulmonary pathologist, and MB) who were blinded to all clinical data. Based on the 2021 WHO classification (15), the diagnoses were revised according to the predominant pattern. All histologic patterns and their percentages, in 5% increments, were recorded.

Some histopathological features known to have prognostic significance, such as angiolymphatic invasion, vascular invasion, tumor diameter, pleural invasion, and lymph node metastasis were also documented.

### Immunohistochemistry

Paraffin blocks that were representative of all histologic patterns present together were selected for immunohistochemical analysis. For this purpose, one to two blocks per case were used. Immunostaining for MUC1, MUC2, MUC5AC, and MUC6 proteins was performed on 4-µm tissue sections, using an automated staining module (Ventana Medical System-Benchmark XT/ISH Staining Module, Roche, Switzerland). Tissue sections were incubated with the primary antibodies with different incubation periods and different dilutions. Anti-MUC1 antibody (Ma695, 1:100, Leica/Novocastra), anti-MUC2 antibody (Ccp58, 1:50, Leica/Novocastra), anti-MUC5AC antibody (CLH2, 1:50, Leica/Novocastra) and anti-MUC6 antibody (CLH5, 1:50, Leica/Novocastra) were used.

### Evaluation of Immunohistochemistry Results

**Figure 1:**
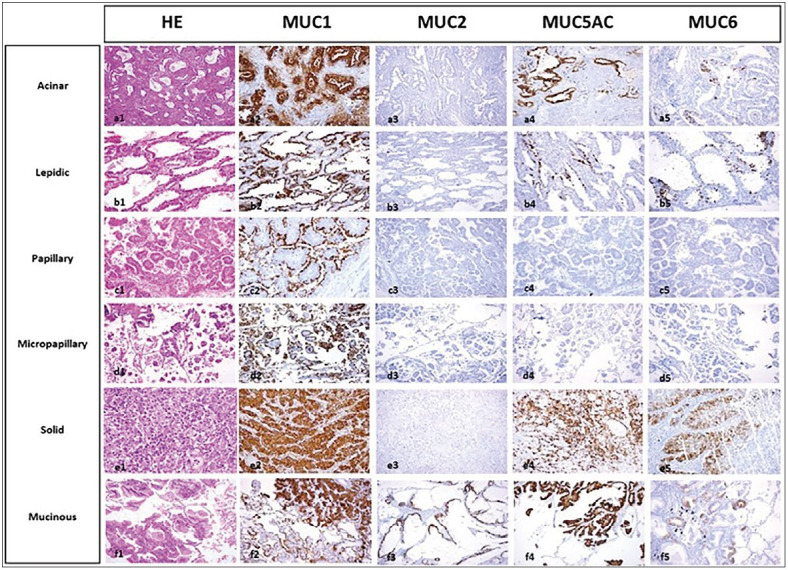
Expressions of MUC1, MUC2, MUC5AC, and MUC6 in various histologic patterns of invasive adenocarcinoma (a-f). The acinar pattern shows MUC1 (depolarized staining), MUC5AC, and MUC6 positivity (a2, a4, a5). MUC2 is negative in this pattern (a3). The lepidic pattern shows polarized staining for MUC1 (b2). MUC5AC and MUC6 are focally positive and MUC2 is negative (b3-5). Papillary and micropapillary patterns show polarized staining for MUC1 (c2, d2). MUC2, MUC5AC, and MUC6 are negative in papillary (c3-5) and micropapillary (d3-5) patterns. The solid pattern shows MUC1 (depolarized staining), MUC5AC and MUC6 positivity (e2, e4, e5). MUC2 is negative (e3). Mucinous adenocarcinoma shows both polarized and depolarized MUC1 expression (f2). MUC2, MUC5AC, and focal MUC6 positivity in mucinous adenocarcinoma (f3-5). (a1, a3, b3, c3, d1, d5, f2, f3 x100; c2, e1, e5 x400; others x200)

**Figure 2:**
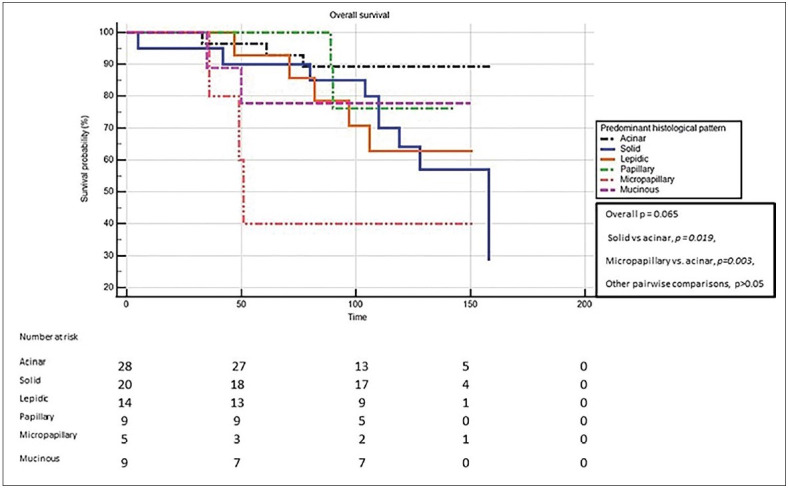
Kaplan-Meier survival analysis for predominant histologic patterns (n=85).

We evaluated the percentages and the intensity of staining for each antibody (MUC1, MUC2, MUC5AC, and MUC6). Cytoplasmic staining was observed for antibodies MUC2, MUC5AC, and MUC6. MUC1 expression was subclassified depending on the expression pattern into ‘polarized’ or ‘depolarized’ expression as follows: (1) polarized expression if MUC1 was localized into the cellular membrane of the apical portion of tumor cells (Figure 1), (2) depolarized if MUC1 was observed over the entire cell surface or whole cytoplasm (Figure 1). In tumors showing both polarized and depolarized expression, if the areas with depolarized expression comprised more than 20% of the tumor, the staining was accepted as depolarized. The pattern of MUC1 staining was further divided into two subgroups for statistical comparisons, one containing negative and polarized stained cases and the other containing depolarized stained cases.

The percentage of positive cells and the staining intensity were recorded for each pattern and scored using a widely used combined scoring system (immunoreactive score, IRS) (28). IRS gives a score between 0 and 12 as a product of multiplication of positive cells proportion score (0-4) and staining intensity score (0-3). According to the IRS, scores of 0-1 are considered negative, scores 2-3 as mild, scores 4-8 as moderate, and scores 9-12 as strongly positive (Table 1).

**Table 1:** The immunoreactive score (IRS).

**A (percentage of positive cells)**	**B (intensity of staining)**	**IRS score (multiplication of A and B)**
0: no positive cells	0 = no color reaction	0-1 = negative
1: <10% of positive cells	1 = mild reaction	2-3 = mild
2: 10-50% positive cells	2 = moderate reaction	4-8 = moderate
3: 51-80% positive cells	3 = intense reaction	9-12 = strongly positive
4: >80% positive cells	**IRS score (A×B): 0-12**	

After calculating IRS scores for each histologic pattern, we calculated a final IRS score (FIRS) per tumor due to the histologic variety of lung adenocarcinomas. Because lung adenocarcinomas contain more than one pattern and the percentage and intensity of staining in each pattern vary, we calculated the FIRS with a formula as follows: (IRS score of *i* pattern x % of *i* pattern) + (IRS score of *ii* pattern x % of *ii* pattern) + (IRS score of *iii* pattern x % of *iii* pattern) + ..... the same formula for any other pattern(s) if present. The final IRS score (FIRS) was further categorized as negative, mild, moderate, and strong, similar to IRS.

For statistical analysis, the cases were divided into two groups depending on the MUC1 staining intensity. Negative (FIRS 0-1) and mild-staining (FIRS 2-3) groups were accepted as group 1, and moderate (FIRS 4-8) and strong-staining (FIRS 9-12) groups were accepted as group 2.

### Statistical Analysis

All statistical analyses were performed using the Statistical Package for Social Sciences (SPSS) software for Windows version 21.0 (IBM Corp, Armonk, NY, USA). The normality of continuous data was checked using both numerical tests (Kolmogorov-Smirnov and Shapiro-Wilk tests) and graphical methods (histogram, Q-Q, and box plots). Data are given as mean and standard deviation (mean ± SD) or median (interquartile range, [IQR]). Categorical variables were compared using Chi-square and Fisher’s exact tests where appropriate. Proportions are given as percentages. Mann-Whitney U and Kruskal-Wallis tests were used for quantitative variables with non-parametric distribution. The Bonferroni correction was used for pairwise comparisons for Kruskal-Wallis test. Significant factors found in univariate tests were included in multivariate analysis. Logistic regression analysis was used for multivariate analysis to identify predictors for lymph node metastasis and the associated risk in terms of odds ratio (OR) and 95% confidence intervals (CI). The Kaplan-Meier test was performed using both SPSS 21.0 and MedCalc statistical software version 20.113 (Ostend, Belgium) for OS comparing survival with the log-rank test and the inclusion of the number at risk table. P-values of less than 0.05 were considered significant in all comparisons.

## RESULTS

### Patient Characteristics

The study included 75 males (75.8%) and 24 females (24.2%). The mean age was 64 (range, 36-84) years. The median tumor size was 3.5 [IQR: 2.5-5] cm. Patients underwent lobectomy (n=83, 83.8%), pneumonectomy (n=5, 5.1%) or wedge resection (n=11, 11.1%). Thirty-nine patients had lymph node metastasis [stage N1: 19 (19.2%), stage N2: 18 (18.2%), stage N3: 2 (2%)]. The median follow-up time was 123 [IQR: 94-141.5] months.

### Histopathological Features

Histopathologic subtypes were as follows: acinar predominant (34.3%, n=34), solid predominant (23.3%, n=23), lepidic predominant (14.1%, n=14), papillary predominant (10.1%, n=10), micropapillary predominant (7.1%, n=7), and invasive mucinous adenocarcinoma (11.1%, n=11). A total of 193 patterns including tumors with more than one pattern (71 acinar, 30 lepidic, 25 papillary, 20 micropapillary, 34 solid, and 13 mucinous) were evaluated. Lymphovascular invasion, venous invasion, and perineural invasion were seen in 62%, 35%, and 15% of 99 adenocarcinoma cases, respectively.

### Immunohistochemical Analysis

Expressions of MUC1, MUC2, MUC5AC, and MUC6 in various histologic patterns are shown in Figure 1. The MUC1, MUC2, MUC5AC, and MUC 6 expression and IRS scores calculated for all histologic patterns are shown in Table 2. Although there were no significant differences regarding MUC1 expression (positive vs. negative) among the histologic patterns, MUC1 IRS scores were significantly higher in lepidic and solid patterns compared to the mucinous pattern (*p=0.006* and *0.0222*, respectively). MUC2 expression was only seen in three cases (1 acinar, 2 mucinous). MUC5AC and MUC2 expression were more commonly seen in mucinous patterns (*p<0.001* and *p=0.028*, respectively), and IRS scores for MUC5AC and MUC2 were higher in the mucinous pattern when compared with the other patterns (*p<0.001* and *p=0.003*, respectively). MUC6 expression was only seen in seven patterns with weak expression. No significant difference was seen among histologic patterns for the staining scores of MUC6.

**Table 2:** MUC1, MUC2, MUC5AC, and MUC6 expressions and average IRS scores by pattern.

**Histologic patterns**	**n=193**	**MUC1+ cases (n)**	**MUC1** **IRS score** **median [IQR]**	**MUC2+** **cases** **(n)**	**MUC2** **IRS score** **median** **[IQR]**	**MUC5AC+ cases** **(n)**	**MUC5AC** **IRS score** **median [IQR]**	**MUC6+ cases (n)**	**MUC6** **IRS score** **median [IQR]**
**Acinar**	71 (36.8%)	61 (85%)	9 [3-12]	1 (1.4%)	0	6 (8.4%)	0	2 (2.8%)	0
**Lepidic**	30 (15.5%)	27 (90%)	10.5 [6-12]	0 (0%)	0	3 (10%)	0	1 (3.3%)	0
**Papillary**	25 (13%)	21 (84%)	9 [2.5-12]	0 (0%)	0	0 (0%)	0	0 (0%)	0
**Micropapillary**	20 (10.4%)	17 (85%)	10.5 [2.2-12]	0 (0%)	0	1 (5%)	0	0 (0%)	0
**Solid**	34 (17.6%)	30 (88.2%)	9 [6-12]	0 (0%)	0	6 (17.6%)	0	2 (5.8%)	0
**Mucinous**	13 (6.7%)	7 (53.8%)	2 [0-5]	2 (15.3%)	0	11 (84.6%)	6 [2-12]	2 (15.3%)	0
**Overall p**		0.123*	0.013**	0.028*	0.003**	p<0.001*	p<0.001 (all)**	0.052	0.051**

*Fisher-Freeman-Halton exact test, **Kruskal-Wallis test Significant p-values are given in bold. **IRS:** Immunoreactive score, **IQR:** Interquartile range

The clinicopathologic features for both group 1 and group 2 of MUC1 expression are summarized in Table 3. No significant difference was detected between the two groups for parameters such as sex, age, tumor stage, tumor size, angiolymphatic invasion, vascular invasion, or stage. There was no difference in MUC1 staining intensity among predominant histologic patterns except mucinous adenocarcinoma, in which MUC1 showed low expression more frequently than in other histologic subtypes (*p=0.01*). High MUC1 expression was also more frequently observed in lymph node-positive cases and cases with perineural invasion (*p=0.003* and *p=0.036,* respectively).

**Table 3:** Relationships between clinicopathologic features and expression of MUC1 in patients with lung adenocarcinoma

	**All patients** **n=99**	**Group 1** **(FIRS 0-1 and 2-3)** **n=21**	**Group 2** **(FIRS 4-8 and 9-12)** **n=78**	**p**
**Age (mean±SD)**	64.5±9.6	66.5±8.9	63.9±9.8	0.269^a^
**Tumor diameter (cm) median [IQR]**	3.5 [2.5-5]	3.8 [2.7-6.1]	3.5 [2.5-5]	0.384^b^
**Follow-up time (month) median [IQR]** n=85	123 [94-141.5]	126 [109-150]	122.5 [90-138]	0.378^b^
**Sex** Male Female	75 (75.8%) 24 (24.2%)	16 (21.3%) 5 (20.8%)	59 (78.7%) 19 (79.2%)	0.958^d^
**T stage** 1 2 3 4	32 (32.3%) 32 (32.3%) 18 (18.2%) 17 (17.2%)	5 (15.6%) 6 (18.8%) 6 (33.3%) 4 (23.5%)	27 (84.4%) 26 (81.3%) 12 (66.7%) 13 (76.5%)	0.51^e^
**N stage** 0 1 2 3	60 (60.6%) 19 (19.2%) 18 (18.2%) 2 (2%)	17 (28.3%) 2 (10.5%) 1 (5.6%) 1 (50%)	43 (71.7%) 17 (89.5%) 17 (94.4%) 1 (50%)	0.059^e^ **N0 vs N≥1 p=0.03^d^**
**TNM stage** (n=98) 1 2 3	35 (35.7%) 28 (28.5%) 35 (35.7%)	7 (20%) 7 (25%) 6 (17.1%)	28 (80%) 21 (75%) 29 (82.9%)	0.742^d^
**Lymphovascular invasion** -Absent -Present	37 (37.4%) 62 (62.6%)	8 (21.6%) 13 (21%)	29 (78.4%) 49 (79%)	0.939^d^
**Venous invasion** -Absent -Present	64 (64.6%) 35 (35.4%)	15 (23.4%) 6 (17.1%)	49 (76.6%) 29 (82.9%)	0.464^d^
**Perineural invasion** -Absent -Present	84 (84.8%) 15 (15.2%)	21(25%) 0	63 (75%) 15 (100%)	**0.036^c^**
**Predominant histologic pattern** -Acinar -Solid -Lepidic -Papillary -Micropapillary -Mucinous	34 (34.3%) 23 (23.2%) 14 (14.1%) 10 (10.1%) 7 (7.1%) 11 (11.1%)	7 (20.6%) 4 (17.4%) 1 (7.1%) 0 3 (42.9%) 6 (54.5%)	27 (79.4%) 19 (82.6%) 13 (92.9%) 10 100%) 4 (57.1%) 5 (45.5%)	0.92^d^ 0.77^c^ 0.289^c^ 0.114^c^ 0.162^c^ **0.01**^c^

**FIRS:** Final immunoreactive score, **IQR:** Interquartile range. Significant p values are given in bold. ^a^Student t-test, ^b^Mann-Whitney U test, ^c^Fisher’s exact test, ^d^Pearson Chi-square test, ^e^Fisher-Freeman-Halton Exact test

The differences between polarized and depolarized MUC1 expression among histologic subtypes are summarized in Table 4. Depolarized MUC1 expression was higher in acinar and solid patterns, whereas polarized expression was seen very frequently in lepidic, papillary, micropapillary, and mucinous patterns (*p<0.001*). We found that depolarized expression was significantly more frequent in acinar and solid patterns when compared with the others (*p<0.001* and *p<0.001*, respectively).

**Table 4:** MUC1 expressions according to histologic patterns.

**Histologic patterns**	**MUC1**			**P**
	**Negative**	**Positive polarized**	**Positive depolarized**	
Acinar (n=71)	10 (14.1%)	12 (16.9%)	49 (69%)	**<0.001**
Lepidic (n=30)	3 (10%)	21 (70%)	6 (20%)	
Papillary (n=25)	4 (16%)	13 (52%)	8 (32%)	
Micropapillary (n=20)	3 (15%)	10 (50%)	7 (35%)	
Solid (n=34)	4 (11.8%)	0 (0%)	30 (88.2%)	
Mucinous (n=13)	6 (46.2%)	4 (30.8%)	3 (23.1%)	
**Total number: 193**	30 (15.5%)	60 (31%)	103 (53.3%)	

Significant p-values are given in bold.

Depolarized MUC1 expression was related to the presence of lymphatic invasion, tumor diameter, and stage. Depolarized expression was detected more common in tumors with lymphatic invasion when compared with tumors without (73% vs. 27%, respectively; *p=0.015*). Tumors with depolarized staining were larger tumors (4.8±3 cm vs. 3.3±1.9 cm, *p=0.013*) and of more advanced stage (*p=0.035*). However, no significant correlation was observed between depolarized MUC1 expression and the presence of vascular invasion (*p=0.724*), perineural invasion (*p=0.313*), or lymph node metastasis (*p=0.135*).

### Univariate tests and Multivariate Analyses for Lymph Node metastasis

We found that tumor size (*p=0.006*), lymphatic invasion (*p=0.018*), vascular invasion (*p=0.025*), perineural invasion (*p=0.019*), mean MUC1 IRS score (*p=0.018*), and MUC1 IRS scores >8.5 (*p=0.018*) were significant predictors for lymph node metastasis (Table 5).

**Table 5:** Univariate tests and multivariate logistic regression analysis of lymph node metastasis.

	**Univariate tests**				**Multivariate analysis**	
	**Lymph node metastasis** **Negative n=60**	**Lymph node metastasis Positive n=39**	**Odds Ratio** **(95% CI)**	**P**	**Odds Ratio** **(95% CI)**	**P**
Tumor diameter cm (mean±SD)	3.7±2.5	5.1±2.8		0.006*	1.207 (1.019-1.431)	**0.03**
Lymphatic invasion -Absent -Present	28 (75.7%) 32 (51.6%)	9 (24.3%) 30 (48.4%)	2.917 (1.184-7.182)	0.018**	1.997 (0.732-5.447)	0.177
Vascular invasion -Absent -Present	44 (68.8%) 16 (45.7%)	20 (31.3%) 19 (54.3%)	2.613 (1.117-6.109)	0.025**	1.604 (0.58-4.44)	0.363
Perineural invasion -Absent -Present	55 (65.5%) 5 (33.3%)	29 (34.3%) 10 (66.7%)	3.793 (1.184-12.147)	0.019**	2.376 (0.614-9.193)	0.21
Predominant histologic pattern -Acinar -Solid -Lepidic -Papillary -Micropapillary -Mucinous	19 (55.9%) 14 (60.9%) 9 (64.3%) 6 (60%) 3 (42.9%) 9 (81.8%)	15 (44.1%) 9 (39.1%) 5 (35.7%) 4 (40%) 4 (57.1%) 2 (18.2%)	-	0.639**		
MUC1 -Negative -Polarized -Depolarized	8 (80%) 23 (67.6%) 29 (52.7%)	2 (20%) 11 (32.4%) 26 (47.3%)		0.156**		
MUC1 -Negative and polarized -Depolarized	31 (70.5%) 29 (52.7%)	13 (29.5%) 26 (47.3%)		0.073**		
MUC1 FIRS scorea (mean±SD)	6.92±4.37	9.01±3.48		0.018*	1.152 (1.023-1.296)	**0.02**
MUC5AC FIRS score (mean±SD)	1.49±3.2	1.06±2.93		0.739*		
MUC6 FIRS score (mean±SD)	0.3±0.95	0.09±0.38		0.133*		
MUC1 FIRS cut-offa ≤8.5 >8.5	33 (73.3%) 27 (50%)	12 (26.7%) 27 (50%)	2.75 (1.176-6.429)	0.018*		

Significant p values are given in bold. a MUC1 FIRS score and MUC1 FIRS categoric groups using 8.5 cutoff were put in two different models * Mann Whitney U test, ** Chi-square test

Tumor size, lymphatic invasion, vascular invasion, perineural invasion, and MUC1 IRS scores indicated that larger tumor size (OR: 1.197, 95% CI: [1.009-1.419]; *p=0.039*) and higher MUC1 IRS scores (OR: 1.814, 95% CI: [1.092-3.013]; *p=0.021*) were independent predictors for an increased risk of lymph node metastasis (Table 5).

### Survival Analysis

There was no statistically significant relationship between sex, T stage, N stage, lymphovascular invasion, venous invasion, perineural invasion, predominant histologic pattern, MUC1, MUC2, MUC5AC, MUC6 expression, depolarized MUC1 staining, and OS according to Kaplan-Meier survival estimates (*p >0.05*). However, solid and micropapillary predominant adenocarcinomas had poor survival compared to acinar predominant tumors (*p=0.003 *and* p=0.019, *respectively) (Figure 2)*.*

## DISCUSSION

We assessed the expression profiles of MUC1, MUC2, MUC5AC, and MUC6 in pulmonary adenocarcinomas in terms of their differences between histologic patterns and their relations with clinicopathologic parameters. We further tested a unique calculation model for immunoreactivity score for MUC1, in which the differences between each histologic pattern were also taken into account (FIRS score). To our knowledge, this is the first report on expression patterns of various MUCs in different histologic subtypes of lung adenocarcinomas. MUC1 FIRS scores may be used as a predictor for lymph node metastasis in addition to conventional parameters such as lymphovascular and perineural invasion.

Previous studies showed controversial results for MUC2, MUC5AC, and MUC6 expression in lung adenocarcinomas (23–26). In a study comparing 7 mucinous adenocarcinomas (previously known as mucinous bronchioloalveolar carcinoma (mBAC)) and 27 non-mucinous BAC, higher levels of MUC2, MUC5AC, and MUC6 expression were found in mucinous BAC (26). In another study, the expression percentages of MUC2, MUC5AC, and MUC6 were higher in mBAC than in solid adenocarcinoma (17% vs. 10%, 97% vs. 21%, and 75% vs. 10%, respectively) (24). Similar results were found for MUC5AC in 10 mucinous adenocarcinomas (mBAC) (100%), but not for MUC2 (0%) in another study (25). Low frequency of MUC2 expression was also demonstrated in another study (only in one of 25 invasive mucinous adenocarcinomas) (23). Our data on MUC2 and MUC5AC expression in mucinous tumors were similar to the literature with low expression for MUC2 (2/13) and high expression (11/13) for MUC5AC. Conflictingly, MUC6 was also low (2/13) in our series. MUC6 expression has been thought to be based on either a metaplastic or a heterotopic presence of gastric mucosa (24). We could not demonstrate any significant MUC6 expression either in mucinous or non-mucinous patterns (acinar, lepidic, papillary, micropapillary, or solid). MUC6 expression was present only in two mucinous and five non-mucinous cases with very low IRS scores. In summary, based on our data, expression of MUC2 and MUC6 was present in a limited number of cases and did not differ among histologic patterns. We think that these two types of MUCs are not useful for histologic subtyping and do not play a role in pulmonary carcinogenesis.

In our study, mucinous adenocarcinoma differed from other histologic subtypes regarding MUC1 and MUC5AC expression. Mucinous adenocarcinoma showed less MUC1 expression with lower IRS scores and higher MUC5AC expression. MUC1 IRS scores were moderate to high in acinar, lepidic, papillary, micropapillary, and solid patterns of non-mucinous adenocarcinomas. The highest MUC1 expression levels were observed in lepidic and solid patterns. Similar to our results, mucinous adenocarcinomas showed apical MUC1 expression in less than 50% of tumors and was more commonly expressed in lepidic predominant adenocarcinomas compared with invasive mucinous adenocarcinomas in the literature (23,26).

MUC1 expression in cancer cells has been shown on both the apical border and the lateral cell membrane and in the cytoplasm (depolarized staining). Both the overexpression of MUC1 and the depolarized pattern of its expression act as a poor prognostic parameter in lung cancer (16–20). Decreased polarized and increased depolarized MUC1 expression was significantly associated with the progression from atypical adenomatous hyperplasia through bronchioloalveolar carcinoma to mixed types (26). Depolarized MUC1 expression and its relationship with poor prognostic parameters such as lymph node metastasis and stage have been well documented among lung adenocarcinomas (19). However, there are conflicting results on the prognostic significance of the depolarized pattern of expression for MUC1 (18,29). Although some researchers demonstrated a relation between depolarized MUC1 expression and early postoperative death (18), no relation between depolarized MUC1 expression and poor survival was found by others (29). In our study, tumors with depolarized MUC1 expression showed lymphatic invasion more frequently and were larger in diameter with advanced stage. However, we found no correlation between depolarized staining and lymph node metastasis or OS. Polarized MUC1 expression was more common in micropapillary and papillary patterns, which are known to have a poor prognosis. Therefore, the presence of depolarized MUC1 expression may be a poor prognostic parameter, but expression levels and interpretation criteria may also be important.

Conflicting results may be due to the lack of standardized immunohistochemical evaluation for MUC1. In previous studies, several criteria for MUC1 immunohistochemical evaluation were introduced (16–18,23,24,26,29). These methods have been used as follows: (a) a four-tiered system depending on the percentage of positive tumor cells (score 0, 0%; score 1, 1-25%; score 2, 26-50%; score 3, 51-75%; score 4, >76%) (24), (b) a four-tiered system depending on the percentage of the polarized or depolarized staining (low-grade polarized, fewer than 50%; high-grade polarized, more than 50%; low-grade depolarized, fewer than 10%; high-grade depolarized, more than 10%) (18,26), (c) a binary system (positive or negative) using the multiplying the percentages of positive-stained cells by the staining intensity and considered positive when the score was ≥10 (17,23), (d) a binary system using a cut-off level (5%) for positivity (16), and (e) a binary system depending on the depolarized expression percentage (positive, >25%; negative, 0-25%) (29). The dominant pattern was used for scoring in another study because tumors showed heterogeneous staining (17). We think that the different criteria for MUC1 immunohistochemical staining are the reasons for such conflicting results. Due to the heterogeneity of pulmonary adenocarcinomas, immunohistochemical stains may show different degrees of staining in each pattern. Therefore, a standard evaluation system is needed for immunohistochemical results to obtain objective data. We tested a final IRS score, in which the differences between each histologic pattern were taken into account. We showed that MUC1 FIRS scores were higher in patients with lymph node metastasis, and tumors with a FIRS score of >8.5 were 2.75 times more likely to metastasize.

MUC1 is overexpressed in lung cancer, making it an excellent target for immunotherapy. Several clinical trials of MUC1 vaccines (L-BLP25) in lung cancer have been reported (21,22). A randomized phase IIb study of L-BLP25 (MUC1) vaccine in stage IIIB and IV NSCLC was conducted looking at survival and toxicity in patients (22). The study showed that the median survival time of patients receiving immunotherapy (88 patients) was 4.4 months longer than that of patients in the control group (83 patients). A phase I trial of the BLP25 (MUC1 peptide) liposomal vaccine in patients with advanced-stage lung cancer established its safety profile and immunogenicity (21). Although the vaccine did not induce a specific humoral response, it did induce cytotoxic T-cell activity in five of 12 patients. There were no objective clinical responses among the 12 patients. In these studies, the intensity of MUC1 expression in the tumor was not taken into account. More promising results can be obtained with new studies in which the degree of MUC1 expression is also considered.

As a limitation, we did not consider the molecular basis of our retrospective study cohort and oncologic approaches affecting prognosis. Nevertheless, our study is one of the few studies examining the differences in mucin expression in lung adenocarcinoma. In addition, we investigated detailed immunohistochemical evaluation according to the patterns and compared them with the prognostic data.

## CONCLUSION

In summary, the present study introduces an alternative scoring for MUC1 that may serve as a predictor for lymph node metastasis regardless of the histologic subtype. Furthermore, MUC1 may be a useful biologic marker for lung adenocarcinoma treatment. MUC1-targeted immunotherapy may be more appropriate for tumors showing high scores of MUC1 expression. Further clinical studies are needed to confirm the role of MUC1 in clinical practice and develop novel therapeutic approaches. We think that our method can also be used to evaluate other immunohistochemical stains used for other heterogeneous tumors to provide objective data collection.

## Conflict of Interest

There are no conflicts of interest.

## Funding

Funding received from the Scientific Research Projects Unit of Istanbul University for this study.
